# Editorial: Novel Strategies for Cancer Immunotherapy: Targeting Immune-Mediated Suppressive Mechanisms

**DOI:** 10.3389/fimmu.2021.691899

**Published:** 2021-05-03

**Authors:** Virginie Lafont, Sophie Lucas, Nathalie Bonnefoy

**Affiliations:** ^1^ Institut de Recherche en Cancérologie de Montpellier (IRCM), Inserm U1194, Univ Montpellier, Institut du Cancer Montpellier (ICM), Montpellier, France; ^2^ de Duve Institute, UCLouvain, Brussels, Belgium

**Keywords:** tumor environment, immunotherapy, immunosuppression, regulatory cells, tumor escape

Over the past decade, novel forms of cancer therapies, collectively referred to as cancer immunotherapies, have emerged and yielded spectacular results, unfortunately in what still remains to date a minority of cancer patients. Immunotherapies mobilize the immune system to promote or restore effective anti-tumor immune responses. Pioneer approaches targeting the CTLA-4/B7 and PD1/PDL1 immune checkpoints (so-called Immune Checkpoint Inhibitors or ICIs) have now broad clinical applications. Although ICIs often offer durable benefits, complete or nearly complete tumor responses occur in only a minority of cases, and resistance is observed in a substantial fraction of patients. Primary or acquired resistance to ICIs are common, with predictive markers of efficacy or resistance remaining difficult to identify. Major efforts are currently made to identify new alternative or complementary targets that activate, unleash or enhance antitumor immune responses.

In this context, developing strategies to target the immunosuppressive tumor microenvironment (TME) is of utmost importance. Current challenges are to decipher at molecular and cellular levels the immunosuppressive mechanisms that most significantly contribute to primary or acquired resistance of cancer cells to anti-tumor immune responses, and provide proof-of-concept that targeting these mechanisms exerts therapeutic anti-tumor activity. A better understanding of the interplay between cancer cells and immune cells is required to develop novel strategies to improve the outcome and increase the proportion of patients responding to cancer immunotherapy. For example regulatory T cells (Tregs), which are important modulators of adaptive immunity and are indispensable to maintain self-tolerance, are well-known to favor local immunosuppression within tumors. Nevertheless, due to the diversity of the immunosuppressive mechanisms used by these cells to exert their functions, the multiplicity of the various Treg subpopulations identified to date, and the existence of many other cell types endowed with immunosuppressive functions, original investigations are required to identify and better define novel therapeutic targets that could help to achieve effective anti-tumor immunity.

The aim of this special issue is to provide an overview of the immunosuppressive mechanisms that seem to prevail within the TME as well as the identification of novel, therapeutically targetable immunosuppressive mechanisms. The various articles illustrate how far the field has advanced, but also remind us of the extent of its complexity.

Thus, in this issue Guo et al. start by reviewing the state-of-the-art on clinical studies and patent applications for PD1/PD-L1 targeted therapies, discussing advantages and disadvantages of new classes of PD1/PD-L1 interaction inhibitors.

Next, many articles concentrate on immune evasion mechanisms as potential new predictive biomarkers of resistance to immunotherapy or targets for novel combination strategies.

Two review articles focus on immunosuppressive cell subsets found in the TME. Chabab et al. describe the mechanisms of action and the role of regulatory γδ T cells, whereas Ngiow and Young summarize key approaches to target immunosuppressive cells such as Tregs and tumor associated macrophages to induce changes in the TME and reinvigorate anti-tumor immune responses.

Metabolic reprogramming of cancer cells is recognized as a well-established hallmark of cancer (1), and emerges as a key immunosuppressive mechanism of the anti-tumor immune response. This topic is reviewed by Mathew and Torres, focusing on the role of lysophosphatidic acid as an inflammatory lipid that binds to the LPA5 receptor on CD8 T cells, as well as by Jacobs et al., describing how 5’-deoxy-5’-methylthioadenosine, an oncometabolite often present in TME, impairs NK cell activity and function.

Several articles or case reports bring interesting data providing rationale for novel combinations to increase ICI efficacy. Guida et al. describe the potential of combining a somatostatin analog with checkpoint inhibitor immunotherapy in Merkel-cell Carcinoma. Qu et al. report that when combined with IL-36, anti-CTLA-4 mAbs increase the proliferation and IFN-γ production by CD4+ and CD8+ T cells and reduce lung metastasis in murine mammary carcinoma, by comparison to single therapies. Burke et al. report benefits of combining histone deacetylase inhibitors with ICIs in bladder cancer. They show that the inhibition of histone deacetylase renders bladder cancer cells more visible, recognizable and destructible by T cells. Ireland et al. investigate the effects of stromal GAS6 protein in pancreatic ductal adenocarcinoma and showed that it alters cancer cell plasticity, activates NK cells and inhibits pancreatic cancer metastasis. Finally, a case report by Shui et al. describes durable responses and tolerance to a triple combination with a novel PD-1 inhibitor plus two chemotherapy agents (Gemcitabine and nab-paclitaxel) in metastatic pancreatic ductal adenocarcinoma. Novel combinations to increase response rates to ICIs are also discussed in three reviews. In the first, Chuang et al. describe the mechanism of action and impact of a monotherapy with TLR-9 agonists and discuss the rationale and the current status to combine them with ICIs as a novel strategy to treat cancer. In a second review, Skeate et al. discuss the effects of LIGHT (Tumor necrosis factor superfamily member 14) delivered or expressed in tumors on the vascular reorganization and generation of tertiary lymphoid structures, and how this can greatly improve immunotherapeutic strategies against cancer. In a third review, Goruganthu et al. discuss the effects of Notch ligand receptor recruitment on anti-tumor immune responses, as well as major clinical and preclinical findings that highlight the potential therapeutic activity of targeting this pathway.

Finally, mining GEO and TCGA databases, Li et al. identify an immune risk signature that could serve as a predictor of survival and immune activity in colon cancer.

Thanks to this collection of articles, this issue provides insightful perspectives on immunosuppressive mechanisms at play in the TME, their impact on anti-tumor immunity, and the identification of novel targets for the immunotherapy of cancer.

## Author Contributions

All authors listed have made substantial, direct and intellectual contribution to the work, and approved it for publications. All authors contributed to the article and approved the submitted version.

## Funding

NB and VL are supported by a public grant overseen by the French National Research Agency (ANR) as part of the “Investissements d’Avenir” program (reference: ANR-10-LABX -53-01), by the “Institut National de la Santé et de la Recherche Médicale” and by University of Montpellier.

## Conflict of Interest

NB is a co-founder of OREGA Biotech.

The remaining authors declare that the research was conducted in the absence of any commercial or financial relationships that could be construed as a potential conflict of interest.
